# Public health and medical preparedness for mass casualties from the deliberate release of synthetic opioids

**DOI:** 10.3389/fpubh.2023.1158479

**Published:** 2023-05-12

**Authors:** Susan M. Cibulsky, Timo Wille, Renée Funk, Danny Sokolowski, Christine Gagnon, Marc Lafontaine, Carol Brevett, Rabih Jabbour, Jessica Cox, David R. Russell, David A. Jett, Jerry D. Thomas, Lewis S. Nelson

**Affiliations:** ^1^Chemical Events Working Group of the Global Health Security Initiative, Public Health Agency of Canada, Ottawa, ON, Canada; ^2^Administration for Strategic Preparedness and Response, US Department of Health and Human Services, Boston, MA, United States; ^3^Bundeswehr Institute of Pharmacology and Toxicology, Munich, Germany; ^4^Bundeswehr Medical Academy, Munich, Germany; ^5^Centers for Disease Control and Prevention and Agency for Toxic Substances and Disease Registry, US Department of Health and Human Services, Atlanta, GA, United States; ^6^Chemical Emergency Management and Toxicovigilance Division, Health Canada, Ottawa, ON, Canada; ^7^Battelle Memorial Institute, Columbus, OH, United States; ^8^Chemical Security Analysis Center, US Department of Homeland Security, Aberdeen Proving Ground, MD, United States; ^9^Chemicals and Environmental Hazards Directorate (Wales), UK Health Security Agency, Cardiff, Wales, United Kingdom; ^10^National Institutes of Health, US Department of Health and Human Services, Bethesda, MD, United States; ^11^National Center for Environmental Health, Centers for Disease Control and Prevention, US Department of Health and Human Services, Atlanta, GA, United States; ^12^Department of Emergency Medicine, Rutgers New Jersey Medical School, Newark, NJ, United States

**Keywords:** chemical incident, emergency preparedness, opioids, disaster, illicit opioids, fentanyl, naloxone

## Abstract

The large amounts of opioids and the emergence of increasingly potent illicitly manufactured synthetic opioids circulating in the unregulated drug supply in North America and Europe are fueling not only the ongoing public health crisis of overdose deaths but also raise the risk of another type of disaster: deliberate opioid release with the intention to cause mass harm. Synthetic opioids are highly potent, rapidly acting, can cause fatal ventilatory depression, are widely available, and have the potential to be disseminated for mass exposure, for example, if effectively formulated, via inhalation or ingestion. As in many other chemical incidents, the health consequences of a deliberate release of synthetic opioid would manifest quickly, within minutes. Such an incident is unlikely, but the consequences could be grave. Awareness of the risk of this type of incident and preparedness to respond are required to save lives and reduce illness. Coordinated planning across the entire local community emergency response system is also critical. The ability to rapidly recognize the opioid toxidrome, education on personal protective actions, and training in medical management of individuals experiencing an opioid overdose are key components of preparedness for an opioid mass casualty incident.

## Introduction

The availability of highly potent synthetic opioids in the illegal drug market in North America and Europe has grown greatly over the past 20–25 years (1–3). This creates opportunity for the deliberate release of such substances to cause a mass exposure incident (4). Synthetic opioids are amenable to special formulation, using certain technical resources and subsequent dissemination via air, food, or water, to cause harm. Since risk is commonly considered to be the product of probability and consequence (5), and greater availability of synthetic opioids increases the probability of their employment in a deliberate attack, it follows that the risk is increased. The higher potency of opioids currently prevalent in the illicit drug market also contributes to increased risk through increasing the consequences on health if an attack were to occur.

The history of opioids development and their medical and non-medical uses have been covered extensively elsewhere (6–9). This paper focuses on recent trends in quantities and types of synthetic opioids being produced, distributed, and consumed for non-medical purposes and their implications for the risk of mass casualty incidents. The importance of community preparedness for this type of incident and considerations specific to synthetic opioids are discussed. A synthetic opioid's high potency, rapid onset of toxicity, and ability to be disseminated (by certain mechanisms such as aerosolization for an inhalational route of exposure, depending on specific technical capabilities) could combine in lethal force. The probability of this type of occurrence is low but not zero. In a well-designed and executed deliberate release, the impact to human health and life would be significant without swift intervention (10, 11).

Lifesaving response requires well-trained responders and astute bystanders to recognize the nature of the incident and then follow quickly with protective actions that will terminate exposure and keep patients ventilated until they receive definitive medical care. Those successful outcomes depend on coordinated planning, preparedness, and response of the affected local emergency response community. The objective of this paper is to serve the ultimate goals of saving lives and protecting health during response and recovery by raising awareness of the risk of and concepts to enhance preparedness for a mass casualty synthetic opioid incident. Findings of a workshop conducted by the Global Health Security Initiative's Chemical Events Working Group with participants from the emergency response community are expanded upon here (7).

## Feasibility of mass casualties from the deliberate release of a synthetic opioid

### Availability

The feasibility of mass casualties resulting from the deliberate release of a chemical is a function of the *availability*, the *potency*, and the *feasibility to deliver* the chemical in a manner that will result in mass harm. Synthetic opioids are widely available in the illicit market (1) due to their ease of synthesis, ease of mixing with other substances, and profitability. Since 2013, the world has witnessed continued growth of opioid overdose deaths and an increase in the proportion of those deaths due to synthetic opioids, mainly attributed to illicitly manufactured fentanyl and fentanyl analogs. The National Center for Health Statistics of the US Centers for Disease Control and Prevention (US CDC) reported that since 2016, overdose deaths involving opioids have been highest for synthetic opioids other than methadone. In 2021, approximately 88% of opioid overdose deaths in the US were attributed to synthetic opioids other than methadone; the age-adjusted rate for this subset of opioid overdose deaths was 22% higher than in 2020 (12). Canada is experiencing a similar problem: from 2016 to 2020 there was a 120% increase in the number of opioid overdose deaths. From January to March of 2022, 85% of the opioid toxicity deaths involved fentanyl (3).

The fentanyl molecule contains four moieties, each of which can be modified while maintaining the basic opioid pharmacological properties (13). Computational chemists at Pacific Northwest National Laboratory estimate that millions of distinct fentanyl analogs could possibly be made (14). The vast possibilities for modifying the fentanyl chemical structure allow illicit suppliers to adapt to new laws, law enforcement strategies, and users' preferences. A large number of new synthetic opioid identifications in drug submissions were documented during the 2010s (7). For example, the US Drug Enforcement Administration (DEA) identified 50 new synthetic opioid compounds in submissions during the years 2015–2017 (7) and in Europe, 28 new fentanyl analogs have been reported since they first appeared in 2012 (15). More recently, new non-fentanyl-related synthetic opioids such as nitazene analogs and brorphine appeared on the illicit market and grew in popularity, as evidenced by the number of identifications in biological samples analyzed at the Center for Forensic Science Research and Education and NMS Labs (16). Increased regulation of fentanyl-related substances by the US and Chinese governments may have played a role in the emergence of non-fentanyl-related opioids on the illicit market but also may have contributed to other changes such as a shift in manufacturing from China to Mexico (17).

The increase in opioid overdose deaths is associated with greater amounts of synthetic opioids flowing in illicit markets, which is reflected in data on drug submissions from law enforcement agencies (18). For example, the quantity of illicit fentanyl seized at US borders increased from 2,800 pounds in 2019 to 11,200 pounds just 2 years later (19). In individual law enforcement actions, seizures of several kilogram quantities of fentanyl or other opioids are common. Illicit opioids are often transported and distributed in white powder form, but fentanyl is also found in counterfeit prescription opioid pills. Increasingly, fentanyl is also discovered in mixtures with other drugs such as amphetamine and cocaine (1).

### Potency

The higher the potency of a drug, the lower the quantity of material necessary, if properly delivered, to produce a given effect. In a study of the ventilatory effects of intravenous (IV) fentanyl in healthy human volunteers, doses above 2.9 mcg/kg produced apnea (20). This suggests that doses in the range of hundreds of micrograms (a very small amount of the pure product) could be lethal through ventilatory depression and consequent hypoxia. Several fentanyl analogs, such as sufentanil and carfentanil, are even more potent than fentanyl (21), potentially putting these chemicals in line with some of the highly potent chemical warfare agents such as nerve agents in terms of their ability to cause illness and death. However, the LD_50_ (dose that would be lethal to 50% of the exposed population) in humans is not known precisely and is influenced by many factors. Most available data on opioid potency are based on analgesic effect. Relative potency (equi-effective dose ratio) among opioids for lethality may differ slightly from relative potency for analgesia, causing differences in therapeutic index (the ratio between lethal and therapeutic dose) (2). The overall result is that increasing the opioid dose to achieve effective analgesia can lead to consequential ventilatory depression, which is the primary lethal effect of opioids.

### Feasibility to deliver

The manufacturing of fentanyl and its analogs is not dependent on agricultural inputs (as with heroin), but rather on the availability of laboratory equipment and chemical precursors, which are much more difficult to detect than fields of opium poppies. Thus, manufacture could be performed in a clandestine laboratory, or the product purchased on the illicit market. Fentanyl salts, which are more common on the street than free base (22), are relatively soluble (23) and resistant to degradation in water (24). Synthetic opioids exist in solid powder form under normal conditions. They can be readily absorbed after inhalation and ingestion but not through the skin unless specially formulated (25). Accordingly, inhalation and ingestion are the routes of exposure most amenable to a mass casualty attack.

Opioids take effect rapidly; after inhalation, loss of consciousness and ventilatory depression can appear in minutes (11). The Moscow theater siege in 2002 demonstrated the feasibility of such an attack (26). Russian authorities disseminated a toxic substance, which evidence suggests was a mixture of remifentanil and carfentanil, through the ventilation system of the theater during a standoff with Chechen rebels who had taken ~900 hostages. Over 120 hostages died and hundreds more were hospitalized (10, 11). The weaponization method in the Moscow theater incident is unknown. Fentanyl has a very low vapor pressure and particles are not readily suspended in the air under normal circumstances, i.e., in the absence of deliberate effort to aerosolize the substance in weaponized form (27). In an oral uptake scenario in which food or drinking water are contaminated, adverse health effects would also manifest quickly—possibly within tens of minutes (28), depending on the opioid dose and the type of food or medium. If the contaminated food or water is ingested at different locations and/or times, then the victims could be widely distributed. The ease of access to large quantities, their high potency, especially among the synthetic varieties, their rapid onset of action, their toxicity, and their ability to be disseminated together make opioids a mass casualty weapon of opportunity.

## Pharmacology and toxicology of opioids

### Mechanism of action

Opioids interact with a family of opioid peptide (OP) receptors to produce their physiological and behavioral effects. The International Union of Basic and Clinical Pharmacology (IUPHAR)-approved nomenclature for the four OP receptors is as follows: μ, mu or MOP; δ, delta or DOP; κ, kappa or KOP; and NOP, for the endogenous ligand nociception or orphanin FQ (29, 30). Opioid peptide receptors are expressed in the central and peripheral nervous systems as well as in neuroendocrine, immune, and ectodermal cells, with each receptor type exhibiting a distinct distribution pattern (31, 32). The receptor types also have varied roles in the range of effects elicited by agonists (e.g., analgesia, sedation, decreased gastrointestinal motility, euphoria, dysphoria, psychotomimetic effects, ventilatory depression, pruritus, dyspnea, miosis, nausea and vomiting, urinary retention, and physical dependence) (33). MOP receptors are the primary mediators of the effects of the most common exogenous opioids, including effects that are clinically sought, those that may be craved in substance use disorders, and adverse events (32). The OP receptors belong to the G-protein coupled receptor superfamily. Through inhibitory G-proteins, they all inhibit cyclic adenosine monophosphate (cAMP) formation, activate G-protein gated potassium channels and inhibit voltage-gated calcium channels. The overall effect is hyperpolarization of target cells and reduced neurotransmitter release at neuronal synapses, neuromuscular junctions, and neuroendocrine junctions (34–36).

### Clinical and non-clinical uses

Due to their clinical effectiveness, opioid therapy has an important role in acute pain management; codeine, fentanyl, methadone, and morphine are included in the WHO Model List of Essential Medicines (37). However, opioids carry considerable potential risk and may not confer long-term benefits for treatment of chronic pain (38). Since morphine was introduced into clinical medicine in the early 1800s, numerous additional opioids have been isolated or synthesized (including natural opiates, semi-synthetic, and completely synthetic compounds), many of which are more potent than morphine. Fentanyl, alfentanil, and sufentanil are administered for analgesia and/or as part of an anesthetic regimen. The advantages of these opioids compared to morphine for anesthesia include more rapid onset of action, greater cardiovascular stability, more favorable adverse event profile, higher therapeutic index or safety margin, and shorter duration of action (39). Some opioids are used to treat cough and diarrhea. Carfentanil is primarily used as a highly potent analgesic agent in large animals by veterinarians and is not intended for therapeutic use in humans. However, carfentanil is used as a radiotracer in positron emission tomography imaging studies in humans. Adverse events associated with therapeutic opioid use include sedation, central nervous system depression, ventilatory depression, bradycardia, hypotension, constipation, urinary retention, pruritus, hives, bronchospasm, nausea, vomiting, and miosis. Clinically, opioids are administered most commonly by oral and IV routes and also can be administered by intramuscular (IM) or subcutaneous (SC) injection, transdermally (dermal patch), transmucosally, intranasally, or sublingually. Persons with opioid use disorder often seek the euphoric and sedative effects of opioids and self-administer them by injection (IV, IM, or SC), ingestion, smoking, or insufflation (40).

### Toxicology

Opioid toxicity manifests as exaggerated physiologic effects of therapeutic opioid use. The classic triad of opioid overdose comprises miosis (pinpoint pupils), ventilatory depression, and depressed level of consciousness (41). This acute toxic syndrome or toxidrome shares some characteristics with the cholinergic toxidrome caused by acute nerve agent or organophosphorus pesticide poisoning and can present a challenge for determining appropriate medical interventions (42, 43). Although this triad of findings are not all consistently present, ventilatory depression is the most serious and predictable consequence of opioid overdose. All aspects of ventilatory activity are lowered, with ventilation rate being the most readily observed. Sustained ventilatory depression can lead to hypoxic brain injury and death (44).

As mentioned, a very small amount of fentanyl (< 1 mg) absorbed systemically can be fatal due to ventilatory depression, making it highly potent as a lethal weapon in a deliberate release scenario. Many other opioids are estimated to have similar or higher potencies compared to fentanyl, although their respective lethal doses in humans are unclear. When inhaled, onset of action of opioids is rapid. One victim of the Moscow theater siege reported losing consciousness within 30 s, and another at least 30 s after first seeing a white, cloudy aerosol in the room but without alarming airway irritation (11). Toxicokinetics (e.g., absorption, onset of action, and duration of effect) following intoxicating doses, may be different from what is expected during therapeutic opioid use (44). Naloxone is a competitive antagonist with high affinity for MOP receptors used to reverse opioid overdose and does not by itself cause ventilatory depression even when administered at high doses. Due to its effectiveness in reversing opioid overdose the drug is widely used by healthcare providers as well as lay people (45).

## Community planning, preparedness, and response

### Challenges

In a hypothetical scenario like the Moscow theater incident of 2002, the decisions, access to resources, and response actions will need to occur rapidly in order to save lives and protect people from becoming ill. Recognition that such an incident is occurring will depend on observations, especially of exposed individuals' signs and symptoms, and any unusual details of the course of events. Since intoxication may occur rapidly (within minutes depending on the dose received by an individual and the route of exposure), field detection technologies are unlikely to play a role; handheld detectors will take time to arrive at the scene in the hands of personnel specially trained to use them. Laboratory analysis of clinical or environmental samples will also not be available in time to inform initial management of patients.

Once suspicion of an opioid mass casualty incident arises, anyone responding will need to take appropriate personal protective measures. The incident may unfold rapidly, making the emergency response dependent on local community resources. This includes any necessary personal protective equipment (PPE) as well as equipment and supplies for managing patients (removal from the scene, decontamination, supportive care, medical treatment) (46, 47). With numerous people becoming ill in a short time, responders may have to triage and prioritize patients until all are able to receive medical care. Large quantities of medical countermeasures, including supportive care devices such as a bag-valve mask and opioid receptor antagonists such as naloxone, may be required (48).

### Planning, preparedness, and response

An effective response to an opioid mass casualty incident will require coordinated, rapid, and efficient actions by the entire local community emergency response system, including law enforcement, emergency medical services, hospitals, public health, poison control, and others, as identified by each local community for itself (49, 50). This requires local organizations to plan and prepare together. Community level risk assessment (51) helps identify and prioritize specific threat agents and scenarios, which in turn guide planning and preparedness. Fortunately, there are commonalities among the many possible chemical threat agents such that preparedness for one facilitates preparedness for others. For example, a rapid onset of clinical effects—seconds to minutes after inhalation—is exhibited by many hazardous chemical agents. Guided by locality-specific risk assessment results as well as the physical properties of commonly available opioids, scenarios can be developed around which education, training, and exercises are conducted and plans and policies established. Law enforcement, the intelligence community, poison centers, emergency medical services, emergency departments, and public health agencies should have a good sense of current, ongoing levels and trends of overdoses and naloxone usage. This ongoing awareness ideally serves as a type of surveillance system, through which unusual spikes in activity, which could indicate a covert deliberate opioid release, may be detected (49). Due to the swiftness with which opioids act and the lack of rapid point of care diagnostic technologies, accurate patient diagnosis and recognition of an opioid-induced mass casualty incident will depend on clinical acumen, keen observation, and awareness of the potential for such an incident by responders (discussed further in the next section).

Stockpiling of responder PPE, supportive care devices such as bag-valve masks, naloxone, and other supplies needs to consider the immediacy with which these items will be required. Emergency responders must either have them upon arrival at the scene or be able to obtain them in short order. Similarly, hospitals may need their normal supplies supplemented for a mass casualty incident (48–50). One potential preparedness mechanism is for hospital pharmacies to maintain extra stock of opioid overdose reversal medication, such as naloxone, that is rotated into regular use before expiration, for cost-efficiency.

Sharing information during the incident is critical. The various organizations within the local response system will need to communicate rapidly and efficiently (49, 50). This involves sharing information about the scene and patient conditions, mobilizing resources to appropriate locations, and distributing patients according to hospitals' capabilities. Communication between responders and people at the scene enables those who were potentially exposed but still ambulatory to take protective actions for themselves. They, as well as bystanders, also may be able to help other people who are incapacitated, by removing them from an unsafe location, making sure their positioning allows them to breathe, performing cardiopulmonary resuscitation, and administering naloxone if it's available. Evidence shows that people are likely to help each other during a disaster (52). In some countries, members of the public are encouraged to learn how to respond to opioid overdose. For example, ensuring an open airway and supporting circulation by chest compressions even without rescue breathing or administering naloxone may be enough to save an individual experiencing an opioid overdose (53).

Crisis communication with the public at large is essential for making them aware of any protective actions they should take for themselves and their families and keeping them updated on the evolving incident. In an incident involving fentanyl or one of its analogs, community members' fears may be particularly strong because of the high potency of these substances. These fears may be compounded by unsubstantiated reports of emergency responders becoming intoxicated during the course of responding to an opioid incident (54). Emergency responders managing patients with opioid overdose are at extremely low risk of fentanyl exposure, either from the environment—as long as they are not in an environment where fentanyl has been deliberately aerosolized—or from the patient and should wear standard PPE (see PPE and Patient decontamination section below). This is because opioids are neither volatile nor well absorbed through the skin (27). The US CDC Crisis and Emergency Risk Communication Program provides a manual, training, and other tools online (55). These communication practices will be most effective if planned for prior to an incident.

## Incident recognition

Because opioids cause intoxication very quickly, responders' judgments are key to recognizing an opioid incident. Further, a rapid point of care diagnostic does not exist and, by and large, ambient air and other environmental media are not regularly monitored for opioids. Toxidromes are defined for the purpose of rapidly identifying the chemical agent category to which a patient has likely been exposed based on the collection of their signs and symptoms to determine appropriate emergency treatment (56). Some findings are common to the opioid and cholinergic toxidromes: miosis and weakness of target organs which can manifest as depression of ventilatory and other (muscular) activity. Therefore, distinguishing between opioid and organophosphorus intoxication requires careful attention to all signs and symptoms. The toxidrome approach is meant to help emergency responders both in the field and at the hospital efficiently evaluate patients and determine a course of treatment. This approach is especially important when environmental and clinical test results are not available.

A mass casualty incident may be either overt (e.g., deliberate release of airborne opioids in an enclosed space crowded with people) or covert (e.g., contamination of food, drinking water, or illicit drugs). In the former case, presenting patients will be concentrated in space and time, making it easier to recognize that an incident has occurred. Still, past incidents have demonstrated that some patients will make their own way to hospitals without being evaluated by emergency medical services and perhaps without hospitals having received information about what has happened at the scene (57, 58). In the latter case, patients will be relatively dispersed in space and time. In all scenarios, but perhaps especially ones with distributed patients, timely recognition of an opioid mass casualty incident depends on communication among organizations throughout the local emergency response system. Clinical observations of multiple patients, possibly in different locations, combined with non-clinical information about the circumstances under which patients became intoxicated will help elucidate the incident. With all participants in the response system (e.g., emergency responders, hospitals, poison control centers, public health authorities) sharing and receiving relevant information, the ability of the system to recognize the incident promptly and accurately will be optimized.

## Personal protective equipment and patient decontamination

In the process of treating individuals experiencing an opioid overdose, hospital-based clinicians and responders in the field ordinarily are at low risk of secondary exposure (27). Nitrile gloves should be worn to prevent the transfer of opioid residue. If powder is visible in the air and/or confirmed or suspected to be aerosolized (note that opioids are not volatile), respiratory and ocular PPE should be worn. As the American College of Medical Toxicology (ACMT) and American Academy of Clinical Toxicology (AACT) note, situations in which there is significant airborne suspension of powdered opioids are unusual (27). The Interagency Board (IAB), the US National Institute for Occupational Safety and Health (NIOSH), and the UK government have made more conservative PPE recommendations for emergency responders that include the use of respiratory protection when any amount of known or suspected opioid product is visible (46, 59, 60). In a well-designed and executed deliberate release of small, aerosolized opioid particles, the risk to responders is greater. This increases the likelihood that respiratory and ocular PPE are warranted, especially for responders managing patients at the scene.

When patients are potentially contaminated with opioid powder or liquid, their clothing (at least outermost layers) should be removed with care not to disturb any powder, for example by cutting off clothing from the upper body instead of pulling it over the head. All potentially contaminated areas of skin or eyes should be washed with copious amounts of water, and soap if available, as soon as possible (25, 47, 61). Bleach solutions should not be applied to skin due to the potential for direct injury to the tissue (61). Patient decontamination protects not only the patient but responders and clinicians managing the patient as well. Assessment of the risk posed by patients due to contamination of their bodies and clothing should inform decisions on the types of PPE to be worn by decontamination team members. Although mass media abound with reports of emergency responders becoming passively intoxicated by opioids in the course of their work, there is no documented credible evidence to support such claims. Responder symptoms reported in NIOSH's health hazard evaluations are not consistent with opioid effects. Laboratory analysis of responders' clinical samples, when conducted, failed to provide evidence of opioid exposure. In an analysis of lay media reports published in North America between January 2012 and March 2018, Herman et al. (54) found that none of the over 1,400 articles they reviewed contained a convincing description of first responder opioid exposure. Their criteria were a plausible route of exposure, symptoms consistent with exposure, and laboratory testing that confirmed exposure. Although fentanyl can be absorbed through the skin, even formulations expressly designed to deliver drug transdermally for pain management have a very slow onset of action, measured in many hours or days (27, 62). This further supports the authors' conclusion that their findings are consistent with the ACMT/AACT position statement (27, 54).

## Medical management

Based on a wealth of direct experience, clinicians recommend treating an individual experiencing an opioid overdose by first supporting ventilation and then administering naloxone starting with a standard dose (see below) followed by escalating doses if the individual does not improve (44, 45, 54). A patient should be positioned in the recovery position to protect and open the airway. Ventilation can be provided with a bag valve-mask if available, or rescue breathing, if appropriate. For administration to an opioid-naive person, a starting adult dose of 0.4–2 mg is recommended in emergent situations (63). Emergency responders may administer naloxone intranasally (IN) or intramuscularly (IM), at conventional doses (e.g., 4 or 0.4 mg, respectively), for ease and speed. Recommended dosing for pediatric patients is 0.1 mg/kg (64). Naloxone dosing is titrated, which is easiest to accomplish via IV administration, to maintain adequate ventilatory function.

Concerns have been raised that individuals experiencing overdose of fentanyl or other higher potency opioids may need larger initial and/or cumulative doses of naloxone (65, 66). Several published reports of opioid overdose outbreaks describe patients needing higher than expected doses of naloxone, and in some cases continuous infusion, due to recurrence of ventilatory depression (48, 50, 67). The majority (if not all) of these cases represent effects of other sedatives or hypercapnia or hypoxia from ventilatory depression, and not unresponsiveness, at a receptor level, to naloxone. At exceptionally high concentrations of fentanyl (not typical in substance use populations), larger doses of naloxone may be required to competitively antagonize at the opioid receptor. Ideally, the same medical countermeasure(s) would fit the purposes of community treatment of individual opioid overdoses, civilian emergency response to a deliberate or accidental mass casualty incident, and even military battlefield use. However, this may be difficult to achieve due to a significantly greater risk of adverse outcome in patients with opioid dependence who receive high dose naloxone. The total dose of naloxone required to treat an overdose patient depends on many factors including the specific opioid agonist and its dose, but conventional dosing as described above is almost always sufficient. In severe cases of opioid overdose, pulmonary edema (i.e., acute respiratory distress syndrome) may be observed, as well as secondary injury to additional organ systems (e.g., renal, hepatic, musculoskeletal) (44).

Since naloxone received its first FDA approval in 1971, the drug has been used widely to treat opioid overdose. Bystander naloxone programs have expanded in recent years. According to a study published by the US CDC, nearly 27,000 overdoses were reversed between 1996 and 2014 with naloxone that had been distributed to laypeople who might witness an opioid overdose (68). The frequency of serious adverse events from naloxone is low; the most common adverse effect is precipitation of withdrawal in people who are physically dependent on opioids (e.g., on prescribed long term opioid therapy). However, although withdrawal can be generally mild, in this population naloxone administration (especially higher doses) can cause pulmonary complications such as acute respiratory distress syndrome (69, 70). In patients known to be physically dependent on opioids, a very small dose of naloxone (0.04 mg IV) is meant to minimize the effects of withdrawal (44, 45). In a mass casualty incident, the percentage of affected individuals with physical dependence on opioids is likely to be small (71). The Biomedical Advanced Research and Development Authority, as part of the Administration for Strategic Preparedness and Response within the US Department of Health and Human Services (HHS), Department of Defense, and National Institutes of Health at HHS are all investigating the possibility of reversing opioid-induced ventilatory depression through mechanisms other than opioid receptor antagonism. This would avoid the precipitation of withdrawal in opioid-dependent individuals and interference with ongoing pain management using opioids (72).

## Remediation and recovery

Once the incident site is delimited, a site-specific cleanup plan incorporating the physico-chemical properties of the specific opioid agent(s), how it was disseminated, and the environmental matrices contaminated, should be established to guide all remediation activities. All applicable laws and regulations must be followed. If visible powder is present, bulk amounts should be transferred to appropriate containers with care not to generate airborne dust. Residue may be removed by dry vacuuming using a recently Dispersed Oil Particulate (DOP)-tested vacuum cleaner with high efficiency particulate air (HEPA) filtration. A negative pressure machine can help reduce dust in the air by removing air from the contaminated area (73, 74).

Detailed site characterization, through environmental sampling, analysis, and risk assessment, should be used to determine which surfaces need decontamination. Water or a detergent solution will physically remove solid opioids from surfaces. Chemical degradation is often proposed as a means to enhance the efficiency and improve the safety of opioid decontamination operations. However, limited research has been conducted, especially in operationally relevant settings, to identify effective methods (75). In a recent study of fentanyl decontamination of non-porous indoor surfaces, water spraying alone (with or without detergent) physically removed 70%−90% of the fentanyl with no evidence of degradation. Peracetic acid and acidified bleach solutions degraded fentanyl on these surfaces while pH neutral bleach and OxiClean™ were less efficacious (76). The solubility of fentanyl decreases above pH 7, which may help explain the reduced ability of the latter two solutions to degrade fentanyl (76). For chemical degradation methods, care should be taken to protect workers from hazards associated with the applied chemicals (i.e., bleach is a respiratory and dermal hazard) and consideration should also be given to potential hazards of degradation products or intermediates.

Health-protective environmental levels have not been established for fentanyl or other opioids, which is challenging for those making decisions on cleanup goals. The state of California recently created a new standard for safe re-occupancy of residential buildings after an incident involving fentanyl or fentanyl analogs (73). The goal was to establish cleanup criteria that are protective of workers' and future residents' health and can be verified through sampling and analysis. State and local health experts relied on fentanyl exposure modeling, previous fentanyl remediation cases, and methods for previous development of a state methamphetamine cleanup standard. Cleanup levels were set at < 0.1 mg/sample for fentanyl and < 0.01 mg/sample for carfentanil, where a sample was obtained by wiping a sterile gauze wetted with 4 ml of methanol across a 100 cm^2^ surface area (73). In a case of contamination of a residential home in Indiana due to a clandestine fentanyl production lab, state and local authorities similarly set the final decontamination level at 0.1 mg/100 cm^2^, which was their lab's lower detection limit (77). To better understand the suitability of these limits, appropriate authorities are encouraged to develop evidence-based health-protective levels. Risk communication remains an essential task during remediation and recovery, as well as during the response phase. Decisions and activities to prepare the site for re-use should be explained to the public openly and transparently by trusted sources.

As with other types of mass casualty incidents involving toxic chemicals, patients with known exposures and those who develop delayed-onset symptoms should receive follow up for clinical evaluation and treatment. Regardless of acute effects, victims of a mass casualty incident may need subsequent behavioral health support. Long-term health effects after recovery from acute opioid overdose have not been adequately documented in opioid naïve people who do not sustain hypoxic neurological injury. New studies may help inform preparedness and response to a future incident.

## Conclusions and recommendations

Synthetic opioids pose risk for mass casualty incidents due to their wide availability, high potency, rapid toxicity, and potential ease of dissemination. Opioids present fast-acting inhalation and ingestion hazards, similar to many other chemical agents. Early recognition of opioid overdose can have great impact, maximizing the opportunity to save lives. However, even an incident with a handful of patients may strain resources, so plans should address surge capacity for opioid overdose reversal medication such as naloxone and other medical supplies, equipment, and personnel. The emergency response community should be made aware of the risks and the required protective actions, and train and exercise for relevant scenarios. To respond rapidly and efficiently, community response organizations should plan and prepare together as a unified system rather than as discrete organizations.

## Author contributions

SC, TW, RF, DS, CG, ML, DR, and DJ conceived of and organized the paper. SC and LN were the primary writers and editors. LN and JT contributed content based on their clinical experience. TW, CB, RJ, JC, and DJ wrote sections of the manuscript. All authors contributed to manuscript revision, read, and approved the submitted version.
